# Supplementary material for: Maternal cortisol is associated with neonatal amygdala microstructure and connectivity in a sexually dimorphic manner
Source: eLife. 2020 Nov 24;9:e60729. doi: 10.7554/eLife.60729 (PMC7685701; doi:10.7554/eLife.60729)
Supplement: Transparent reporting form [file elife-60729-transrepform.pdf]

Westbrook Centre, Milton Road
Cambridge CB4 1YG
UK
P 01223 855340
W elifesciences.org
T @elife

eLife's transparent reporting form

We encourage authors to provide detailed information *within their submission* to facilitate the interpretation and replication of experiments. Authors can upload supporting documentation to indicate the use of appropriate reporting guidelines for health-related research (see EQUATOR Network), life science research (see the BioSharing Information Resource), or the ARRIVE guidelines for reporting work involving animal research. Where applicable, authors should refer to any relevant reporting standards documents in this form.

If you have any questions, please consult our Journal Policies and/or contact us: editorial@elifesciences.org.

Sample-size estimation

- You should state whether an appropriate sample size was computed when the study was being designed
- You should state the statistical method of sample size computation and any required assumptions
- If no explicit power analysis was used, you should describe how you decided what sample (replicate) size (number) to use

Please outline where this information can be found within the submission (e.g., sections or figure legends), or explain why this information doesn't apply to your submission:

The study constituted entirely novel data in terms of the method used and the populations it was applied to, so it was not possible to perform an accurate power calculation prior to data collection.
We therefore estimated sample size using studies reporting relationships between prenatal maternal plasma cortisol and relevant neuroimaging / functional outcomes in mother-child dyads:
1. Maternal plasma cortisol and amygdala volume at 7 years – N=65. Buss et al. PNAS 2012.
2. Maternal plasma cortisol and sexual dimorphism in brain structural connectivity of offspring at 6-9 years – N=49. Kim et al Cerebral Cortex 2017.
3. Maternal plasma cortisol and neonatal amygdala functional connectivity and behavioural outcome, noting sexual dimorphism – N=70. Graham et al Biol. Psych 2019.

Replicates

- You should report how often each experiment was performed
- You should include a definition of biological versus technical replication
- The data obtained should be provided and sufficient information should be provided to indicate the number of independent biological and/or technical replicates
- If you encountered any outliers, you should describe how these were handled
- Criteria for exclusion/inclusion of data should be clearly stated
- High-throughput sequence data should be uploaded before submission, with a private link for reviewers provided (these are available from both GEO and ArrayExpress)

Please outline where this information can be found within the submission (e.g., sections or figure legends), or explain why this information doesn't apply to your submission:

eLife Sciences Publications, Ltd is a limited liability non-profit non-stock corporation incorporated in the State of Delaware, USA, with company number 5030732, and is registered in the UK with company number FC030576 and branch number BR015634 at the address Westbrook Centre, Milton Road Cambridge CB4 1YG, UK | March 2019

Due to the nature of our study populations (peripartum women and newborn infants), maternal hair samples and neonatal brain MRI were each collected on one occasion for the following reasons:

3cm of maternal hair for cortisol concentration *at the time of birth* was required to reflect chronic HPA at the time of delivery, and ethical considerations associated with cutting hair for research demanded we take the minimal quantity required to measure hair cortisol concentration. Maternal hair was cut close to the scalp, at the posterior vertex, and stored in aluminium foil at -20°C. The proximal 3cm of hair were analysed by liquid chromatography-tandem mass spectrometry (LC-MS/MS). See Materials and Methods, page 12.

Due to the fragile nature of human newborns and risks of physiological instability in the MRI environment, neonatal MRI data was collected during a single imaging session on a Siemens MAGNETOM Prisms 3T MRI clinical scanner (Siemens Healthcare Erlangen, Germany) using a 16-channel phased-array paediatric head and neck coil, at the Edinburgh Imaging facility, Royal Infirmary of Edinburgh. We acquired 3D T1-weighted MPRAGE (T1w); 3D T2-weighted SPACE (T2w) and axial dMRI. dMRI was acquired in two separate acquisitions: the first acquisition consisted of 8 baseline volumes (b = 0 s/mm^2 [b0]) and 64 volumes with b = 750 s/mm^2, the second consisted of 8 b0, 3 volumes with b = 200 s/mm^2, 6 volumes with b = 500 s/mm^2 and 64 volumes with b = 2500 s/mm^2; an optimal angular coverage for the sampling scheme was applied. See Materials and Methods, page 12.

102 infants were eligible. Of these, 2 preterm infants died, 12 did not complete the MRI protocol or images were discarded due to movement artefact; 1 had an incidental structural anomaly detected at MRI; and 9 withdrew before MRI scan. This left 78 mother-infant dyads for analysis (Page 4)

No data were excluded for outlier reasons.

There was no high-throughput sequence data in this study.

Statistical reporting

- Statistical analysis methods should be described and justified
- Raw data should be presented in figures whenever informative to do so (typically when N per group is less than 10)
- For each experiment, you should identify the statistical tests used, exact values of N, definitions of center, methods of multiple test correction, and dispersion and precision measures (e.g., mean, median, SD, SEM, confidence intervals; and, for the major substantive results, a measure of effect size (e.g., Pearson's r, Cohen's d)
- Report exact p-values wherever possible alongside the summary statistics and 95% confidence intervals. These should be reported for all key questions and not only when the p-value is less than 0.05.

Please outline where this information can be found within the submission (e.g., sections or figure legends), or explain why this information doesn't apply to your submission:

Hair samples were analysed by liquid chromatography-tandem mass spectrometry (LC-MS/MS), at Dresden Lab Service GmbH (Dresden, Germany), using an established protocol (Gao et al *J Chromatogr B Analyt Technol Biomed Life Sci*.2013). Adult hair commonly grows at 1cm/month so we chose to sample 3cm segments to represented maternal HPA axis activity over the last three months of pregnancy.

We used an optimised neonatal MRI acquisition protocol, summarised in Materials and Methods, page 12 of the manuscript, and described in detail in Boardman et al BMJ Open 2020.

Image data were processed according to the developing Human Connectome Project (dHCP) minimal processing pipeline (Makropoulos et al NeuroImage 2018). The M-CRIB atlas is age-specific for neonatal parcellations (M-CRIB, Alexander et al NeuroImage 2017) and subsequent processing used methods that have been applied successfully to neonatal MRI data (Materials and Methods, Page 13-14).

Individual participant data were analysed using Pearson's correlation. Associations between maternal HCC and image features were investigated using multiple linear regression models, where image feature was the dependent variable and covariates included gestational age at birth, gestational age at scan, SIMD2016, and birthweight z-score. A coefficient of determination, standardised beta coefficient, and False Discovery Rate adjusted p-value, is reported for each analysis. False discovery rate was used to adjust p values for multiple tests. Statistical procedures are described on page 15.

(For large datasets, or papers with a very large number of statistical tests, you may upload a single table file with tests, Ns, etc., with reference to sections in the manuscript.)

Group allocation

- Indicate how samples were allocated into experimental groups (in the case of clinical studies, please specify allocation to treatment method); if randomization was used, please also state if restricted randomization was applied
- Indicate if masking was used during group allocation, data collection and/or data analysis

Please outline where this information can be found within the submission (e.g., sections or figure legends), or explain why this information doesn't apply to your submission:

Randomisation was not applicable to the study design.
Maternal HCC measurement was blinded to case:control status.

Additional data files ("source data")

- We encourage you to upload relevant additional data files, such as numerical data that are represented as a graph in a figure, or as a summary table
- Where provided, these should be in the most useful format, and they can be uploaded as "Source data" files linked to a main figure or table
- Include model definition files including the full list of parameters used
- Include code used for data analysis (e.g., R, MatLab)

- Avoid stating that data files are "available upon request"

Please indicate the figures or tables for which source data files have been provided:

All data generated or analysed during this study are included in the manuscript and supporting files.
Source data upon which figure 2 is based is also presented in supplementary table 2.
